# Sanitary Officers and the Law

**Published:** 1896-05-02

**Authors:** 


### Sanitary Officers and the Law.

A case has recently occurred at Dorchester which shows well the tendency too often displayed by sanitary officials to strain the law for their own purposes—good purposes, no doubt, but beyond the letter of the law. Application was made before the magistrates for an order for the removal to hospital of three children said to be suffering from scarlet fever, and without proper accommodation. The medical officer of health, however, when called, was unable to state what rooms there were, and relied on a statement made to him some time before by the medical man in attendance, to the effect that the children were without proper accommodation. It was suggested by the defence, and was not denied, that there were six rooms in the house, two of which were spare ones, and that, in fact, there was plenty of accommodation; and then it appeared that the real reason why the case was brought forward was that the parents would not keep the children in one room, but allowed them to run about the house. But the Public Health Act says nothing about the distribution of infection inside a private house. What has to be proved is that there is not proper lodging and accommodation, and herein the medical officer failed. The view taken by the magistrates clearly was that lack of nursing and of discipline is not enough, the accommodation itself must be proved to be defective. The medical officer was asked whether a child suffering from an infectious disease and running about a house among its own family, and no person from outside going near it, ought to be removed to a sanitary hospital? He replied in the affirmative, pointing out that the child would be exposing itself and rendering other people in the house liable to infection. This may be so, but it is not in the Act. The medical officer also stated that the children could not have had proper care because they had been nursed by the mother who had been carrying about her baby in her arms. "That is a fine way of nursing scarlet fever cases!" he said. No doubt it is all very wrong, but there is nothing about it in the Act, and until Parliament chooses to legislate as to the nursing of fevers, we fear that medical officers cannot interfere, except in tendering advice. In any case, they must not strain the law. It ultimately appeared that the root of the matter was the fact that the father was a tailor, and that he was doing his work in a room just opposite to the one in which the sick children lay. Clearly this was very bad, and probably the man was amenable to law, but however possible it might be to close the workshop, its presence did not bring the case under the clause relied upon for compelling the removal of the children. We mention this case not because of its intrinsic importance, for a decision before a local bench of magistrates is of no binding legal authority, but as illustrating a tendency, too commonly observable among sanitary officials, to use such legal powers as fall into their hands for the promotion of what they take to be the general objects and aims of sanitary legislation, without being over particular as to the exact meaning of the clauses on which they rely. There is but little doubt that if the parents of these children at Dorchester had not obtained legal assistance a removal order would have been issued—much, doubtless, to the benefit of the town in which they live, but still contrary to the provisions of the Public Health Act.

